# Penile Calciphylaxis: Two Clinical Cases and a Literature Review

**DOI:** 10.7759/cureus.87276

**Published:** 2025-07-04

**Authors:** Jorge Martin Millet, David Josue Saavedra, Antonio Esqueda-Mendoza, Eduardo Cruz Nuricumbo

**Affiliations:** 1 Department of Urology, Hospital Regional de Alta Especialidad de la Peninsula de Yucatan (HRAEPY), Merida, MEX

**Keywords:** diabetes mellitus(dm), necrosis, partial penectomy, penile calciphylaxis, penile diseases, total penectomy, vascular calcification

## Abstract

Penile necrosis secondary to calcification of medium- and small-caliber arteries supplying the organ is a rare and serious condition. It often occurs in patients with multiple comorbidities, carries a high mortality rate, and has a poor prognosis.

We present two cases of male patients with multiple comorbidities who developed penile calciphylaxis. Both patients underwent various treatments, including total penectomy in one case and palliative management in the other. One patient declined surgery and was lost to follow-up, while the other required multiple surgical procedures due to infectious complications.

A review of the literature suggests no clear survival benefit between surgical and conservative management. Therefore, surgical treatment should be reserved for cases with disabling pain or advanced infections at risk of severe complications.

## Introduction

Penile calciphylaxis is an uncommon condition that can occur due to circulatory disorders secondary to the calcification of medium- and small-caliber arteries, especially when associated with metabolic disorders such as diabetes mellitus, end-stage chronic kidney disease, and arterial hypertension. The diagnosis is clinical, based on medical history and physical examination. Mortality is as high as 64%, occurring in 1% of patients with chronic kidney disease [1], and in up to 6% of diabetic patients with end-stage renal disease [2]. The most common symptoms include pain, urinary obstruction, and ulcerative lesions along the penis. Treatment is determined by the patient’s symptoms and overall condition and may be either conservative or surgical.

## Case presentation

Case #1

We present the case of a 58-year-old male patient with a five-year history of type 2 diabetes mellitus with poor treatment adherence; stage 5 chronic kidney disease secondary to diabetic nephropathy, managed with hemodialysis via an arteriovenous fistula for four years; arterial hypertension; and subclinical hypothyroidism, among other conditions. He presented to the emergency department with a 30-day history of a painful, non-secretory, odorless ulcerative lesion with a whitish base, approximately 5 × 5 mm in size, located on the glans penis. He denied engaging in high-risk behaviors for sexually transmitted infections. The patient had previously been evaluated by dermatology, where Behçet’s disease was suspected. He was treated with prednisone 20 mg for 10 days, which led to a reduction in lesion size. However, he returned for follow-up 15 days later with signs of recurrence. He was subsequently prescribed topical terbinafine, copper sulfate, fluconazole, cephalexin, and acyclovir for suspected mixed balanitis and genital herpes.

**Figure 1:**
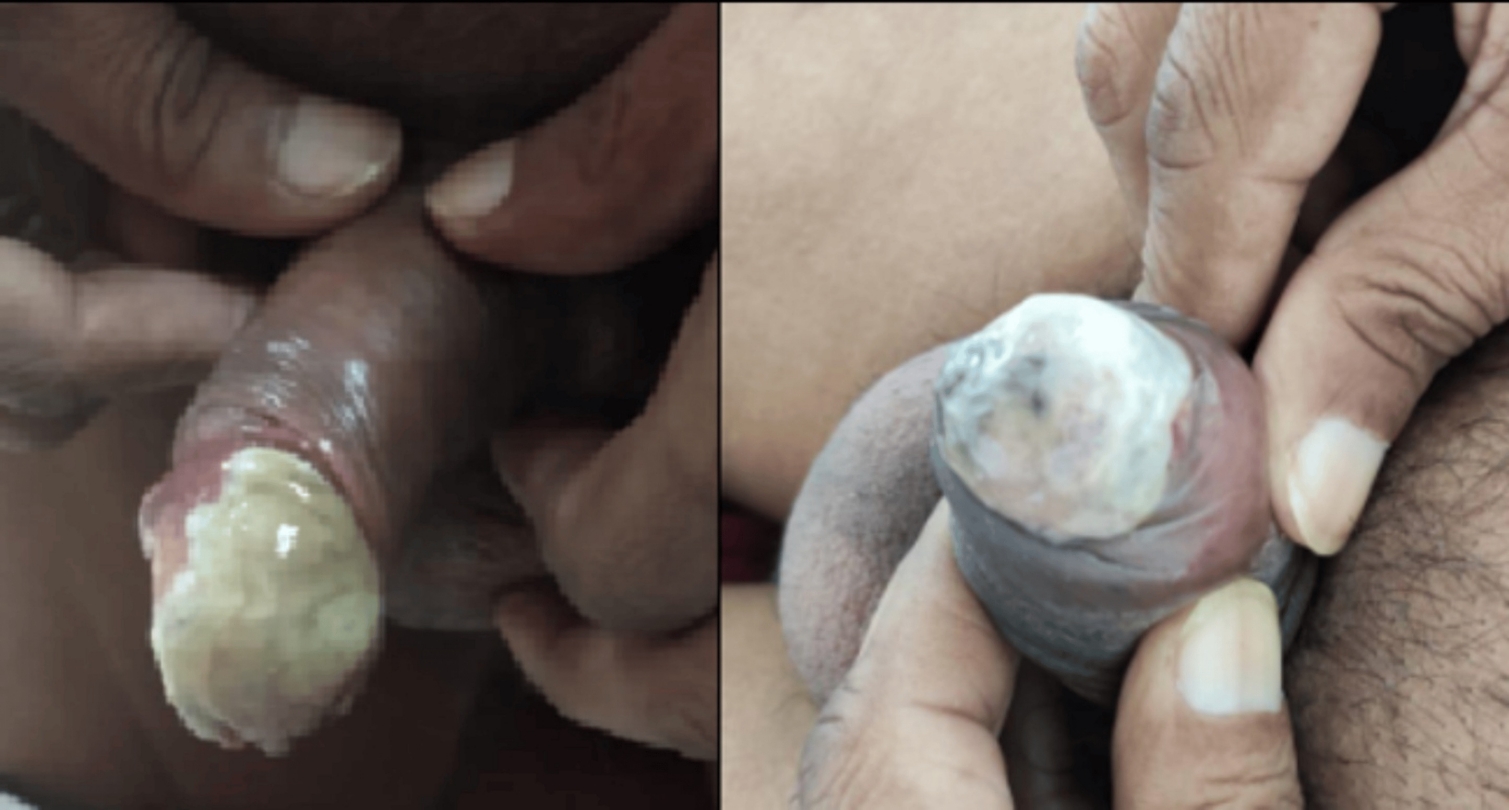
Whitish lesion on the glans with a periurethral necrotic background.

Upon evaluation in the emergency department, the patient appeared cachectic and had palpable, indurated bilateral superficial inguinal lymph nodes. A painful yellow-green plaque infiltrating the deep tissues occupied the entire glans, measuring approximately 20 × 20 mm, with foul-smelling discharge and apparent necrotic tissue on the tip and dorsal surface of the glans (Figure 1).

Serological tests for syphilis (Venereal Disease Research Laboratory (VDRL) test) and HIV were non-reactive. Smear and exudate cultures were positive for Enterococcus faecalis, Enterobacter cloacae complex, and Klebsiella oxytoca. As a result, treatment with piperacillin/tazobactam adjusted for renal function was initiated. After five days of antibiotic therapy without clinical improvement, a penile wedge biopsy was performed, obtaining two samples: one from the foreskin and another from the balanopreputial fold.

**Figure 2:**
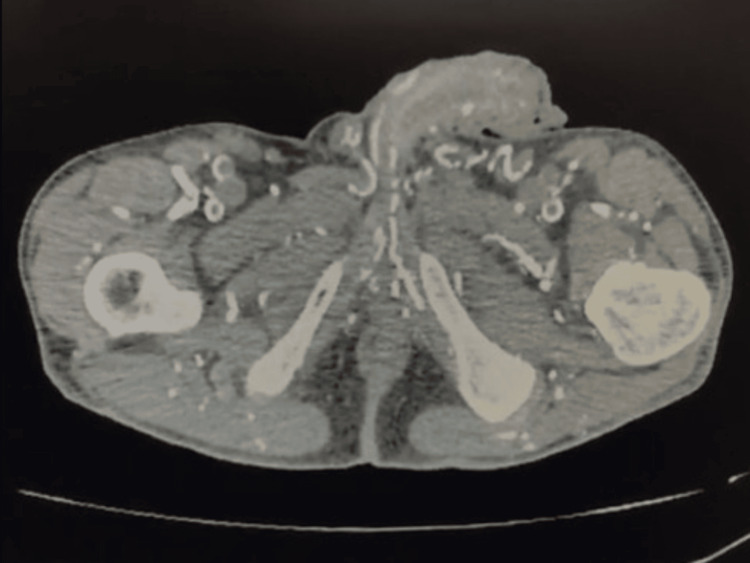
Simple CT scan (axial section) showing extensive calcification of the pelvic and genital vessels.

A computed tomography (CT) scan of the thorax, abdomen, and pelvis revealed calcified atheromatosis of the coronary vessels and thoracic aorta, along with diffuse atheromatous calcification of the intra-abdominal, retroperitoneal, intrarenal, pelvic, and genital vessels (Figure 2).

In light of these findings, the Vascular Surgery team performed a lower limb ultrasound, which revealed extensive calcifications along the entire arterial pathway. Blood flow was preserved in the macrocirculation; however, evidence of microcirculatory arterial damage was observed. As a result, anticoagulant and vasodilator therapy was initiated.

**Figure 3:**
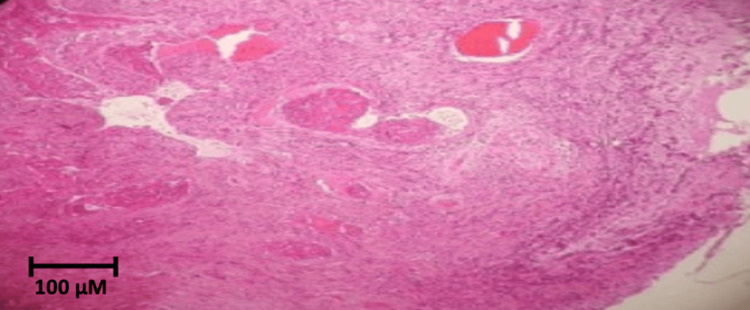
Case #1: Penile biopsy. Histology of the penile biopsy specimen showing diffuse necrosis and granular calcification throughout the soft tissues (hematoxylin and eosin stain, 10×).

The biopsy revealed calcific uremic arteriolopathy with ischemic necrosis (Figure 3), confirming the diagnosis of calciphylaxis. 

Given the progression of the condition and the patient’s clinical deterioration, partial phallectomy was proposed. However, the patient declined the procedure and requested voluntary discharge. He died seven months later without follow-up.

Case #2

We report the case of a 40-year-old Hispanic male with a 19-year history of insulin-dependent type 2 diabetes mellitus with good treatment adherence; stage 5 chronic kidney disease on dialysis for four years; arterial hypertension; diabetic neuropathy; and other comorbidities.

In February 2023, he began experiencing pain at the tip of the penis and was treated with antibiotics. One month later, he presented to our center with severe, agonizing pain, but no additional symptoms. The decision was made to admit the patient for pain control, antibiotic therapy, and evaluation for possible surgical intervention.

**Figure 4:**
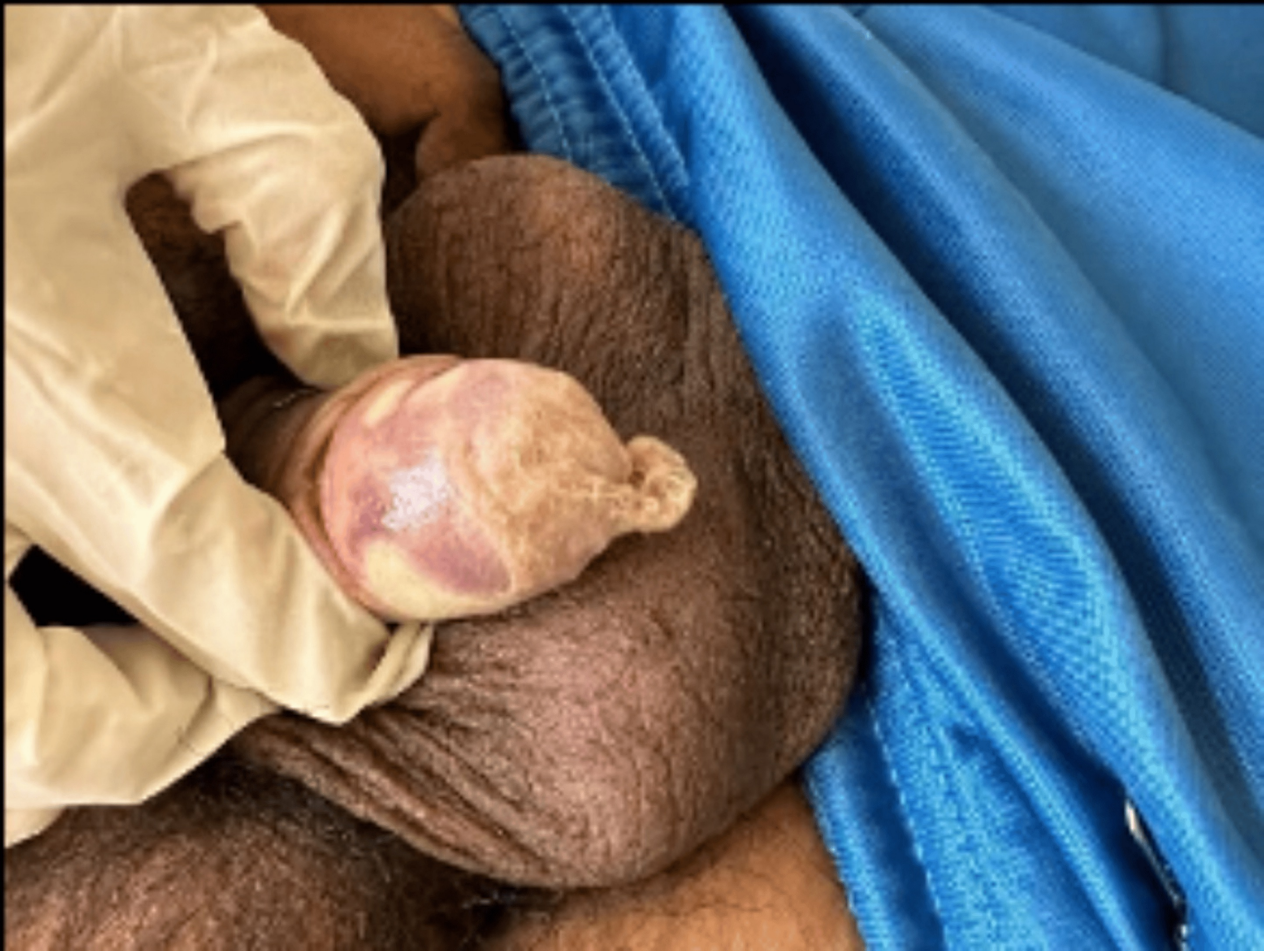
Penis with a sessile lesion on the glans.

On physical examination, an arteriovenous fistula was noted in the left arm; the abdomen was unremarkable. There was an infracondylar amputation of the left lower limb and amputations of the first and fifth toes of the right foot. The penis showed a sessile lesion on the glans, approximately 7 mm in size, with a urethral meatus measuring 3 mm, and no signs of discharge, odor, or inflammation (Figure 4). 

**Figure 5:**
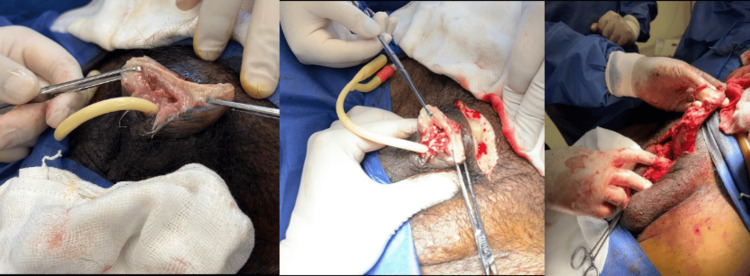
Penectomy with neoglans reconstruction.

During hospitalization, the patient underwent a partial penectomy with neoglans reconstruction without complications (Figure 5) and was treated with ceftriaxone. He was discharged following clinical improvement.

Seven days post-operation, the patient presented with a burning sensation in the neoglans region, accompanied by foul-smelling brown discharge and progressive edema at the base of the penis, extending into the suprapubic area. Additionally, he experienced intermittent fevers over the preceding five days, with no relief from analgesics. Due to suspicion of soft tissue infection extension, hospitalization was indicated for management of the infectious focus.

On physical examination, there was increased volume at the base of the penis extending into the suprapubic region. The area was tender to palpation, indurated, mobile, and euthermic. Postoperative changes consistent with partial penectomy were observed, with the neoglans visible. The penis exhibited a mucocutaneous appearance with foul-smelling brown discharge and a whitish plaque at the urethral meatus, without signs of active bleeding. Both testicles were present in the scrotum without apparent abnormalities.

In the following hospital days, smear and exudate cultures were positive for Serratia liquefaciens, for which antibiotic therapy with imipenem was administered for 14 days. Surgical management was undertaken, including total penectomy and surgical debridement, with no complications.

**Figure 6:**
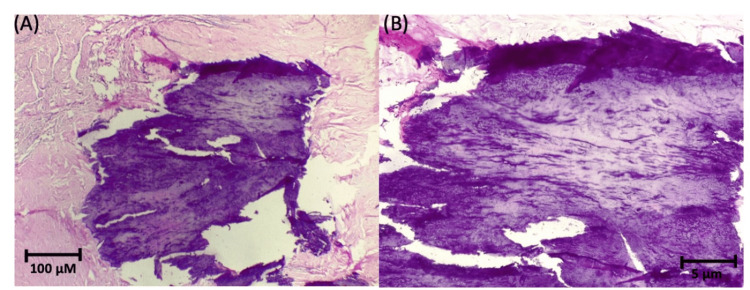
Case #2: Penile biopsy. Histology of the penectomy specimen showing intimal calcification and necrosis: (A) hematoxylin and eosin stain, 10×; (B) hematoxylin and eosin stain, 20×.

Intraoperative findings included necrosis of both corpora cavernosa, purulent exudate extending to the pubic region, necrosis of the distal urethra, and abundant devitalized tissue surrounding the corpora cavernosa. Biopsy findings revealed calcific uremic arteriolopathy with ischemic tissue necrosis (Figure 6), supporting the diagnosis of calciphylaxis. The patient was discharged following clinical improvement.

The patient was admitted for a third time due to foul-smelling purulent discharge from the suprapubic surgical wound and severe, agonizing pain, without other notable symptoms. An urgent transfer to the operating room was made for wound irrigation and debridement to control the infectious focus.

During hospitalization, a second wound irrigation and debridement were performed, and a dual antibiotic regimen with imipenem and cilastatin was initiated for six days. After completing antimicrobial therapy, the patient was discharged for maximum benefit and at his request, despite the associated risks and complications. He later died from acute fulminant heart failure.

## Discussion

Penile necrosis secondary to calcification of medium- and small-caliber arteries is a rare pathology, with few cases reported in the literature. It is most commonly associated with end-stage chronic kidney disease [3,4] and may lead to amputation of affected tissues or limbs [5]. The prognosis is poor, with an overall mortality rate of approximately 64% and an average time from diagnosis to death of less than one year [6,7]. Histopathologically, it is characterized by calcification of small- and medium-sized vessels, fibrosis, thrombus formation, and tissue necrosis [4,7-9].

Risk factors contributing to the development of this condition include chronic kidney disease, diabetes mellitus, hyperparathyroidism, and metabolic disturbances such as bone mineral disorders, calcium-phosphorus imbalance, administration of vitamin D, and prolonged dialysis periods [10-15].

The clinical presentation varies and may include erythema, purplish discoloration, ulceration, and ultimately necrosis of the penile tissue. Previous studies have reported that these lesions tend to occur more commonly in extragenital regions, particularly the lower extremities, making diagnosis challenging when penile lesions are presented in isolation [9,16].

Laboratory findings often show elevated levels of serum calcium, phosphorus, and parathyroid hormone, which support the diagnosis of calciphylaxis [1,9], although these values may remain within normal ranges in certain cases [10]. Routine biopsy is discouraged due to the risk of secondary infection and progression to wet gangrene [17]. Imaging, particularly CT, has proven useful in diagnosis [18]; the presence of a net-like calcification pattern is reported to have 90% specificity [19].

Before initiating treatment, the literature emphasizes the importance of a multidisciplinary approach, involving urologists, nephrologists, vascular and reconstructive surgeons, pathologists, palliative care providers, and dermatologists [20].

Due to its rarity, there is no standardized treatment protocol for penile calciphylaxis. Conservative treatments such as sodium thiosulfate and hyperbaric oxygen therapy have been described. Surgical options reported in the literature include internal iliac artery stenting, revascularization procedures, penectomy, and parathyroidectomy, especially in cases involving calcium metabolism abnormalities [1,8,9,15,21,22].

Current evidence supports a more conservative approach involving wound care, pain control, and systemic therapy aimed at reducing calcium and phosphate levels using calcium-free phosphate binders, along with limiting calcium-based products in patients undergoing dialysis [4,9,15,21].

Among emerging therapies, hyperbaric oxygen plays a role by increasing tissue oxygenation and promoting wound healing through angiogenesis stimulation, fibroblast proliferation, and collagen expression [8].

The surgical management of secondary hyperparathyroidism remains controversial. A 2003 review reported a higher survival rate in patients with systemic calciphylaxis following parathyroidectomy; however, the difference was only eight months and did not reach statistical significance. A later systematic review found no association between parathyroidectomy and mortality outcomes [8].

Another debated treatment for penile calciphylaxis is partial versus total penectomy. Karpman et al. [7] reported no statistically significant difference in survival between patients treated with penectomy and those managed with local debridement and wound care. This finding was later supported by Yang et al. in a 2018 review, which found mortality rates of 42.9% in surgically treated patients and 52% in those receiving conservative treatment [9]. These findings, combined with the generally high mortality rate and a mean survival of 3.4 months in penectomized patients compared to four months in those managed conservatively [1], suggest that surgical intervention does not significantly alter the natural course of the disease. It may increase the risk of postoperative complications and lead to significant psychological distress in penectomized patients [9].

Nevertheless, Barthelmes et al. [6] argued in their 2002 review that early aggressive debridement may improve survival. Overall, the literature does not support penectomy as a first-line treatment. It is reserved for select cases, such as those with intractable pain or advanced infection with a risk of progression to severe sepsis, where surgery may be necessary for survival [8,9,20,23,24].

Penile calciphylaxis is a rare and poorly understood entity. Montoya et al. [25] reported in a retrospective study of 14 cases that the most common initial symptom was a penile eschar, followed by purulent discharge, inflammation of the glans and foreskin, and priapism. In that study, 57% of patients underwent total penectomy, and among them, 37.5% died.

## Conclusions

Penile calciphylaxis may present as a late manifestation of systemic vascular disease and requires a multimodal approach involving various specialties, often before urological evaluation is considered. Although only a limited number of cases have been reported in the literature and no standardized treatment guidelines exist, current evidence favors minimally invasive strategies. Conservative management should be prioritized, given the low survival rates, with an emphasis on improving the patient’s quality of life.

## References

[1] David R, Nowicki J, Lee J, Dean N (2019). Penile gangrene due to calciphylaxis: a multidisciplinary approach to a complex clinical challenge. BMJ Case Rep.

[2] Bolio-Laviada FM, Zamora-Varela FR, Carvajal-García R (2014). Necrosis de pene por calcifilaxis en paciente nefrópata. Rev Mex Urol.

[3] Brandenburg VM, Cozzolino M, Ketteler M (2011). Calciphylaxis: a still unmet challenge. J Nephrol.

[4] Ceccato T, Bruniera M, Iafrate M, Dal Moro F (2023). Penile calciphylaxis, infrequent complication with bad prognosis: a case report. Urol Case Rep.

[5] Naik BJ, Lynch DJ, Slavcheva EG, Beissner RS (2004). Calciphylaxis: medical and surgical management of chronic extensive wounds in a renal dialysis population. Plast Reconstr Surg.

[6] Barthelmes L, Chezhian C, Thomas KJ (2002). Progression to wet gangrene in penile necrosis and calciphylaxis. Int Urol Nephrol.

[7] Karpman E, Das S, Kurzrock EA (2003). Penile calciphylaxis: analysis of risk factors and mortality. J Urol.

[8] McCarthy JT, El-Azhary RA, Patzelt MT (2016). Survival, risk factors, and effect of treatment in 101 patients with calciphylaxis. Mayo Clin Proc.

[9] Yang TY, Wang TY, Chen M, Sun FJ, Chiu AW, Chen YH (2018). Penile calciphylaxis in a patient with end-stage renal disease: A case report and review of the literature. Open Med (Wars).

[10] Gabel C, Chakrala T, Shah R (2021). Penile calciphylaxis: a retrospective case-control study. J Am Acad Dermatol.

[11] Nigwekar SU, Thadhani R, Brandenburg VM (2018). Calciphylaxis. N Engl J Med.

[12] Nigwekar SU, Bhan I, Turchin A (2013). Statin use and calcific uremic arteriolopathy: a matched case-control study. Am J Nephrol.

[13] Price PA, Williamson MK, Nguyen TM, Than TN (2004). Serum levels of the fetuin-mineral complex correlate with artery calcification in the rat. J Biol Chem.

[14] Nigwekar SU, Zhao S, Wenger J, Hymes JL, Maddux FW, Thadhani RI, Chan KE (2016). A nationally representative study of calcific uremic arteriolopathy risk factors. J Am Soc Nephrol.

[15] Hayashi M, Takamatsu I, Kanno Y, Yoshida T, Abe T, Sato Y (2012). A case-control study of calciphylaxis in Japanese end-stage renal disease patients. Nephrol Dial Transplant.

[16] Swatesutipun V, Benyakorn T (2022). Challenges management in penile calciphylaxis. Urol Case Rep.

[17] Sampathkumar SS, Veerappan I, Raman RS, Chakravarthy T, Siddharth VA (2023). Penile calciphylaxis - a rare, yet medically treatable disease. Indian J Nephrol.

[18] Cimmino CB, Costabile RA (2014). Biopsy is contraindicated in the management of penile calciphylaxis. J Sex Med.

[19] Morrison M, Merati M, Ramirez J, Cha HC, LaFond A (2016). Penile calciphylaxis diagnosed with computed tomography. J Eur Acad Dermatol Venereol.

[20] Shmidt E, Murthy NS, Knudsen JM, Weenig RH, Jacobs MA, Starnes AM, Davis MD (2012). Net-like pattern of calcification on plain soft-tissue radiographs in patients with calciphylaxis. J Am Acad Dermatol.

[21] Bkiri S, Tlemsani Z, Khdach Y, Nmili Y, Bennani K, Abbad F, Ghadouane M (2022). Penile calciphylaxis in a patient with end-stage renal disease and chronic hemodialysis: a case report. Pan Afr Med J.

[22] Saito T, Mima Y, Sugiyama M (2020). Multidisciplinary management of calciphylaxis: a series of 5 patients at a single facility. CEN Case Rep.

[23] Adler K, Flores V, Kabarriti A (2021). Penile calciphylaxis: a severe case managed with partial penectomy. Urol Case Rep.

[24] Hussein ZN, Bapir R, Fakhralddin SS, Abdullah AM, Salih KM, Kakamad FH (2023). Penile calciphylaxis with penoscrotal necrosis: a case report with literature review. Urol Case Rep.

[25] Montoya MG, Otero GJM, López SV (2005). Necrosis de pene: Experiencia en el Hospital de Especialidades Centro Médico Nacional Siglo XXI. Bol Col Mex Urol.

